# Should Protocol Kidney Biopsies be a Part of Routine Post-Transplant Care? PRO

**DOI:** 10.34067/KID.0000000000000419

**Published:** 2024-08-13

**Authors:** George T. John, Brian P. Doucet

**Affiliations:** 1 Royal Brisbane and Women's Hospital, Herston, Queensland, Australia; 2 University of Queensland, Queensland, Australia; 3 School of Clinical Medicine, University of Queensland, Queensland, Australia

**Keywords:** transplant pathology, transplantation

Renal allograft biopsies are the gold standard for the identification of underlying pathological processes in the allograft despite the risk of complications, sampling errors, and variations in interpretation.^1^ The interobserver variability of allograft biopsy reporting has decreased with the universal adoption of the BANFF criteria.

In a systematic review of 40,000 renal allograft biopsies, graft loss and mortality were <0.01% and the need to intervene for complications was 0.89%.^2^ The benefits of the prognostic and diagnostic information obtained with a biopsy exceed the risks, especially with a high pretest probability of a treatment-altering result.

Internationally, several transplant centers have implemented protocol biopsies (PBs) as standard practice; however, widespread acceptance of PB is yet to occur not only to be informed of subclinical pathology that may alter the immunosuppressive management and future performance of the allograft but also to validate the noninvasive assessment of allograft.

Renal allograft survival is currently above 80% at 5 years. Short-term graft outcomes have improved with the evolution of standard immunosuppressive regimes; however, longer term graft outcomes have not kept pace, with alloimmunity implicated as the underlying culprit. Subclinical alloimmunity because of inadequately titrated immunosuppression can lead to interstitial fibrosis, tubular atrophy *de novo* donor-specific antibody formation, allograft dysfunction, and premature allograft loss.^3^ Subclinical rejection (SCR) rates have declined considerably from the time of cyclosporine to that of tacrolimus,^1^ but are still significant in recipients monitored with PBs. It is uncertain whether the alteration in treatment when SCR is identified leads to improved graft outcomes. There is an unmet need for long-term, large, multicenter studies for evidence in support of PBs and intervention trials stemming from it.

## Procurement Biopsy

Procurement biopsies are performed on kidneys before transplantation with the view of assessing suitability for transplantation. Centers in the United States that regularly perform these biopsies are currently discarding kidneys at three-fold rates compared with centers that use other variables to determine kidney suitability.^4^ Development of a standardized method of procurement biopsy interpretation, which incorporates histopathologic and molecular determinants using whole-slide reading and artificial intelligence, would likely reduce kidney discard rates.^3^ Procurement biopsies, if trialed and protocolized in this context, could potentially serve to increase organ utilization.

## PB at and after Transplantation

The incidence of SCR with a regimen containing tacrolimus and mycophenolate, with or without steroids, varies from 2.6% to 25% within the first year.^5^ Zero to 1-hour biopsy after transplantation delineates baseline features of glomerulosclerosis, interstitial fibrosis, tubular atrophy, reperfusion injury, underlying glomerular pathology, and the initial immunological reaction. A biopsy at 3 months informs of the presence or absence of rejection. At 1 year, a PB is predictive of long-term allograft survival.^6^

## SCR

High-risk patients with a history of sensitization, previous transplants, and recurrent disease; those patients who have steroid avoidance and calcineurin inhibitor–free protocols; and those who require reduction of immunosuppression for toxicity or side effects should be monitored with PBs because all these scenarios increase the pretest probability of significant subclinical pathology. Increased cold ischemia time and delayed graft function may also increase the risk of SCR. The rate of subclinical antibody-mediated rejection in the first few weeks after transplantation has been reported at 3.3% with higher rates in HLA- and blood groups A,B,O-incompatible transplants.^7^

There is some evidence suggesting early subclinical tubulitis and interstitial inflammation in transplanted kidneys have adverse clinical implications.^5,8^ When SCR is found, it affects allograft survival and potentially benefits with treatment.^5,9^ Early diagnosis and treatment of SCR may not only improve short-term renal allograft function but may also help preserve renal structure and long-term function. To improve long-term transplant outcomes, it is necessary to focus on both invasive and noninvasive diagnostics and on interventions that alter outcomes if SCR is present. SCR in high immunological risk situations could have different behavior and prognosis as compared with that found in low-risk situations. The difference may lie in differential gene expression.^10^

## Development and Validation of Biomarkers from Allograft Biopsies

Biomarkers and molecular diagnostic testing, including microarray testing, are yet to be proven superior to biopsy. These are best used in combination with biopsy findings. There is molecular heterogeneity between acute and SCR. For rigorous evaluation of noninvasive tests, the gold standard of tissue-based diagnostics with PB is essential.^11^ Development and validation of a SCR score could improve the detection of SCR.

Microarray analysis of kidney allograft biopsy tissue has also been used to predict the risk of fibrosis and allograft loss. The commercially available tissue-based molecular microscope diagnostic system discriminates between rejection and nonrejection. Transcriptomic analysis of the kidney allograft is performed using RNA sequencing while NanoString technology can supplement histological interpretation. Urinary exosomal studies have compared favorably with histological T-cell–mediated rejection. AlloSure by CareDx and Prospera by Natera detect donor-derived cellfree DNA in blood, and Trugraf by Transplant Genomics detect differentially expressed genes. These, with further validation, will initially improve yield from directed PBs and are likely to replace PBs in patients with a predefined matrix of risk.

## Summary

When allograft function is stable in a low-risk patient, reluctance to biopsy is not uncommon and is understandable. However, in a high-risk individual and in a high-throughput center for transplantation, PBs have a favorable risk–benefit ratio. While SCR is treated with modifications in immunosuppressive management by most centers, practices vary. In high-risk situations, PBs are advised until noninvasive correlates have clinical validation.

Profiling a dynamic and varied immune response is only feasible with relatively noninvasive diagnostics, preferably a panel of tests with urine and blood. Whole-genome transcriptome profiling seems promising for the unique and shared gene signatures both for T-cell–mediated rejection and antibody-mediated rejection. Clinical validation of such tests would be possible only with information gained from PBs and indicated biopsies.

PBs are essential for monitoring and maintaining allograft health in high-risk kidney transplant recipients. PBs remain the gold standard for validation of noninvasive testing and will provide tissue for deeper insights into allograft injury with molecular and genetic interrogation.
